# Environmental impact scenarios of organic fraction municipal solid waste treatment with Black Soldier Fly larvae based on a life cycle assessment

**DOI:** 10.1007/s11356-023-27140-9

**Published:** 2023-05-02

**Authors:** Navarro Ferronato, Riccardo Paoli, Francesco Romagnoli, Gianluca Tettamanti, Daniele Bruno, Vincenzo Torretta

**Affiliations:** 1 Department of Theoretical and Applied Sciences, University of Insubria, Via G.B. Vico 46, 21100 Varese, Italy; 2 Institute of Energy Systems and Environment, Riga Technical University, Āzenes iela 12/1, Riga, LV-1048 Latvia; 3 Department of Biotechnology and Life Sciences, University of Insubria, Via J.H. Dunant 3, 21100 Varese, Italy; 4 Interuniversity Center for Studies on Bioinspired Agro-Environmental Technology (BAT Center), University of Napoli Federico II, Via Università 100, 80055 Portici (NA), Italy

**Keywords:** Solid waste management, LCA, Circular economy, Sustainable development, BSF, *Hermetia illucens*

## Abstract

Biowaste treatment with Black Soldier Fly (BSF) larvae is an alternative option for organic waste valorization. Its environmental impacts should be assessed and compared with conventional treatment options. The research aims to evaluate the treatment of organic fraction of municipal solid waste (OFMSW) with BSF larvae through a life cycle assessment (LCA). This study employed data inventories from literature and aimed to provide a wide range of production parameter values to identify the potentialities of BSF treatment in the best-case and worst-case scenarios. The SimaPro9, the database Ecoinvent3.5, and the impact assessment method IMPACT 2002+ have been employed for the analysis. A sensitivity analysis of relevant parameters was conducted, considering the avoided impacts that can be obtained thanks to the exploitation of larvae proteins for bioplastics or fishmeal production. Research findings highlight six main environmental impact indicators: respiratory inorganics (kg PM2.5-eq), ozone layer depletion (kg CFC-11-eq), terrestrial ecotoxicity (kg TEG soil), land occupation (m^2^ organic arable), global warming (kg CO_2_-eq), and non-renewable energy (MJ primary). The most relevant process generating impacts is BSF breeding, followed by boiling, storage, and OFMSW treatment. The environmental performance is better when the conventional fishmeal substituted, thanks to BSF larvae production, is made from areas 10,000 km far, implementing a 100% renewable energy scenario, reducing the energy consumption by 50%, increasing the lifespan of the equipment to 15 years, and products are employed locally. The current study represents the first attempt to evaluate the global higher or lower environmental impact scenario related to OFMSW treatment through BSF larvae.

**Graphical Abstract:**

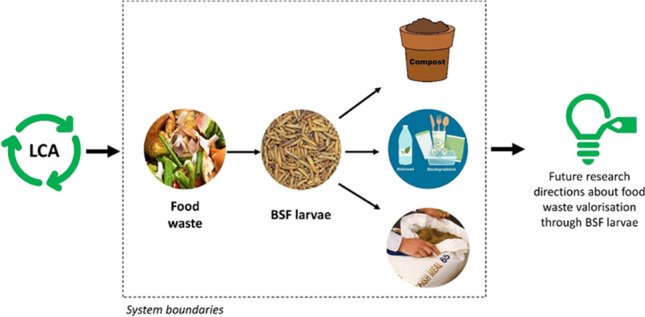

## Introduction

The scientific community is active in evaluating the best options for treating the organic fraction of municipal solid waste (OFMSW) (De Medina-Salas et al. 2019; Logan and Visvanathan 2019). The objective is to improve treatment technologies, find alternatives to valorize food waste, and implement circular economy waste treatment solutions (Mak et al. 2020). Conventional treatment options like composting and anaerobic digestion are not always easy to implement due to economic, environmental, and technical issues (Pergola et al. 2018; Awasthi et al. 2020; Kumar and Samadder 2020). In addition, these treatment options could not be appropriated for developing countries or isolated areas (Surendra et al. 2014; Thomas and Soren 2020). The efforts that engineers and scientists are carrying out are related to assessing the involvement of insects and other invertebrates as alternatives for food waste degradation (Girotto and Cossu 2019).

The choice of the most suitable insect is a key factor, and several characteristics must be considered to find the species that provides the best biodegradation performance (Čičková et al. 2015; Ferronato et al. 2020b). One of the most valid alternative is represented by *Hermetia illucens*, known as Black Soldier Fly (BSF). The BSF larvae (BSFL) are able to reduce the waste volume and give an economic value to the final product being the larva itself, or a derivative, classified by the end-products in terms of fertilizer, protein, energy, or animal feed (Joly and Nikiema 2019). Therefore, BSFL demonstrate to be an alternative option for treating OFMSW due to its simplicity, low maintenance expenses, saving of waste volumes, and emissions (Singh and Kumari 2019).

The selection of this technique instead of others, like composting and anaerobic digestion, also looks promising for the management of downstream usage. The literature agrees that BSFL can reduce nutrient leakage, obtain higher conversion efficiency for biodiesel production, inactivate pathogens (Gorrens et al. 2021), and reduce the spread of odours, thanks to short processing time, and reduced bacterial activity (Joly and Nikiema 2019; Smetana et al. 2019). In particular, from BSFL and pupae, it is possible to obtain proteins and lipids (Cappellozza et al. 2019; Spinelli et al. 2019), which can be employed for feed formulation and biofuel production, respectively, as well as chitin and its derivative chitosan, which can be employed in different fields (Lin et al. 2021). Moreover, BSF proteins have been recently considered for the production of bioplastics (Barbi et al. 2019). Considering that bioplastics play an important role in mitigating the impacts of plastic pollution and the depletion of abiotic resources, finding alternative sources of proteins can be promising and of interest to the international scientific audience (Tettamanti et al. 2022).

Although the use of BSF for the bioconversion of organic waste is exponentially increasing and various companies deal with the commercialization of BSF-derived products, there is still room for improving biobased production valorization and the scalability of the treatment plants. As a new research area, the studies available in the literature are still at the pilot stage (Lopes et al. 2020; Isibika et al. 2021), and even fewer are the studies on the environmental impacts produced by a full-scale BSF process (Ites et al. 2020). The current research aims to provide a comprehensive study related to the environmental impacts generated by a BSF treatment process, indicating the most important environmental impact indicators (EII) and the processes that can affect the environmental benefits obtained by the process.

The life cycle assessment (LCA) method is employed within this study to identify the main global environmental impacts generated by the treatment system within defined boundaries. This approach has been employed in other studies on BSF applications. For example, the use of three by-products as growing substrates for *H. illucens* larvae has been assessed, showing that the production of BSF for the hen diet resulted in the most impact for most of the environmental categories, due to the inclusion of soybean meal in the diet (e.g., 5.79 kg CO_2_ eq per kg of dry larvae) (Bava et al. 2019). Another research evaluated the production of fishmeal from *H. illucens* larvae and *Lemna minor*, underlying that fishmeal based on these insects can be more sustainable than standard feed (Goyal et al. 2021). A study conducted in Indonesia assessed BSF waste treatment facility’s global warming potential (GWP), highlighting that direct CO_2_-eq emissions are 47 times lower than the emissions from composting (Mertenat et al. 2019). However, articles on the LCA of the bioconversion of the OFMSW by BSFL are still limited. In addition, these studies do not allow identifying the main sensitive parameters, the most relevant processes, and the most relevant environmental impact indicators that can potentially affect the environmental benefits given by the whole supply chain.

The LCA conducted and presented in this paper takes advantage of the data available in the literature, elaborating these databases introduced within the inventory analysis to provide a comprehensive analysis of the BSF treatment system. The research questions that deserve an answer are “what is the range of values of environmental impact indicators related to the best and worst case scenario?” and “which parameters, indicators and processes should be carefully analysed to improve environmental benefits of OFMSW”? The study’s objective and novelty are to provide to the international audience a comprehensive assessment of the parameters already available in the literature to add insights related to the processes and products that can be the focus of future research activities about food waste valorization through BSFL. Furthermore, the results obtained by the LCA represent environmental performance thresholds for engineers, biotechnologists, and chemists involved in studying the best outputs that BSFL can generate at laboratory scale.

## Methods

### Research methodology

**Fig. 1:**
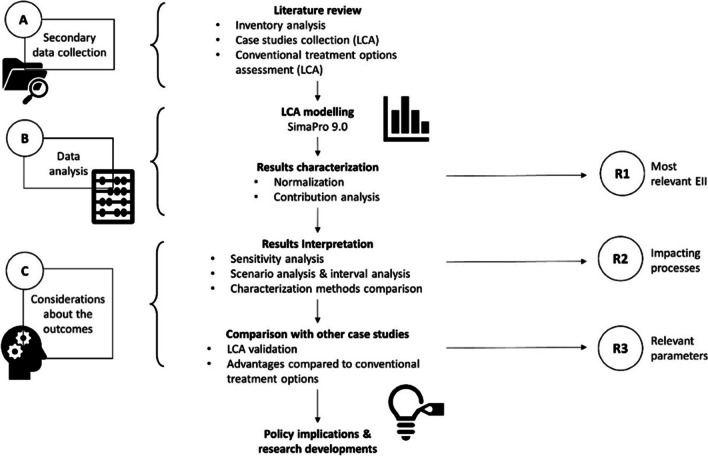
Scheme of the research procedure

The steps followed for implementing the current research are presented in Fig. 1. As reported in the scheme, the study is divided into three main parts: (a) a literature review, where datasets available for implementing the inventory of the LCA were gathered; (b) LCA modelling for the assessment of environmental performance through selected EII; (c) the analysis of the results through a sensitivity analysis, an interval analysis, and the comparison with the results of LCA conducted for assessing the environmental performance of conventional treatment options.

The rationale of the research and the implementation of the LCA is the need to find the processes that can potentially affect the environmental benefits related to the treatment of OFMSW by BSFL, create a clear summary of the results for all the case studies available in the literature before June 2020, and give a comprehensive range of values for the most important EII related to the BSFL treatment options. Three key research questions were addressed in this study:

1. Which EII is mostly relevant to be assessed and considered;
2. Which process mostly affects the OFMSW treatment by BSFL;
3. Which parameter mostly affects the results (sensitivity of the results due to the change of modelling parameters);

The literature review allowed the collection of secondary data and the selection of the parameters that should be potentially assessed. The sensitivity analysis, utilizing the sensitivity ratio (SR), was employed to evaluate the most relevant parameters that affect the results, while the interval analysis from the data collected from the literature aimed to define the maximum and minimum values of the selected range of the parameters. This approach provides the spectrum of the highest and lowest possible impacts that the BSF treatment system can generate, making results comparable with outcomes of other LCA studies.

### Literature review and secondary data collection

Data were gathered through a literature review. Research articles and reports were collected from Scopus® and Google Scholar. The documents utilized refer to a time interval of maximum 10 years. Keywords like “Life Cycle Assessment” AND “blac soldier fly” AND “organic waste” and related acronyms were employed for the analysis. Finally, four scientific articles specifically related to the treatment of OFMSW with BSF and only related to study containing inventory data were retrieved. Table 1 reports the general characteristics of the studies considered for the inventory analysis. Data collected from the selected documents were normalized to the functional unit (FU), thus to a specific unit of measurement. In particular, kilograms per FU were employed for the material balance, while megajoules were employed for the energy balance. The FU used is 1 t of OFMSW.

**Table 1:** List of the literature collected for BSF inventory analysis

Authors	Country	Document	Description of the analysis conducted	LCA software and impact assessment method	EII
Smetana et al. (2019)	Germany	Scientific article	LCA to evaluate the environmental impact with historical production data from the pilot plant and sensitivity analysis about the industrial progress for possible improvements.	• SimaPro8.2 and Ecoinvent 3.1 database. • IMPACT2002+.	All mid- and end-point impact categories from IMPACT2002+.
Dortmans et al. (2017)	Switzerland	Handbook	Description of the practical tasks and materials needed for the treatment of OFMSW by BSF.	• No LCA studies included.	-
Mertenat et al. (2019)	Switzerland	Scientific article	LCA assessment approach to evaluate the GWP of a BSF waste treatment facility in an Indonesian case study and comparison with an open wind-row facility.	• SimaPro8 and Ecoinvent 3.1 database. • GWP (100a) from IPCC 2013	GWP (100a) as GHG emissions (i.e. CO_2_-eq)
Salomone et al. (2017)	Italy	Scientific article	LCA assessment approach to evaluate the environmental impacts related to insect-based products through a treatment of FW by BSF (Southern Italy). Comparison with other conventional feed production systems. (i.e., soybean and rapeseed).	• SimaPro8 software and Ecoinvent 2.0 database. • CML 2 baseline 2000 and GWP (100a) from IPCC 2007 v. 1.	Photochemical oxidation; terrestrial ecotoxicity; marine aquatic ecotoxicity; fresh water aquatic ecotoxicity; human toxicity; ozone layer depletion; IPCC GWP 100a; eutrophication; acidification; abiotic depletion.

### LCA — goal and scope definition

The LCA study was focused on a hypothetical OFMSW treatment plant, which involves OFMSW bioconversion through BSFL at a pilot scale. The goals of the LCA are to (i) identify the most relevant EII of the whole process, (ii) highlight the process that predominantly affects the OFMSW treatment by BSFL, and (iii) define the main parameters that mostly change the results. The reference ISO 14044:2006 Standards (ISO 2006a, b) were considered within the study. The LCA was performed using the SimaPro9.0 software (Goedkoop et al. 2008) and Ecoinvent 3.5 (Wernet et al. 2016). The IMPACT 2002+ was chosen as the impact assessment method (Jolliet et al. 2003).

As mentioned, the FU refers to 1 t of OFMSW and implements a cradle-to-gate approach. All data inputs were normalized considering the treatment of about 365 t of OFMSW per year (i.e., 1 t per day), including the construction materials. According to the IMPACT 2002+ method, 15 mid-point categories were considered. The results at end-points categories (also called eco-profile) are in terms of eco-points representing the average impact per person per year. The consistency of the results was evaluated through a sensitivity analysis and scenario analysis.

**Fig. 2:**
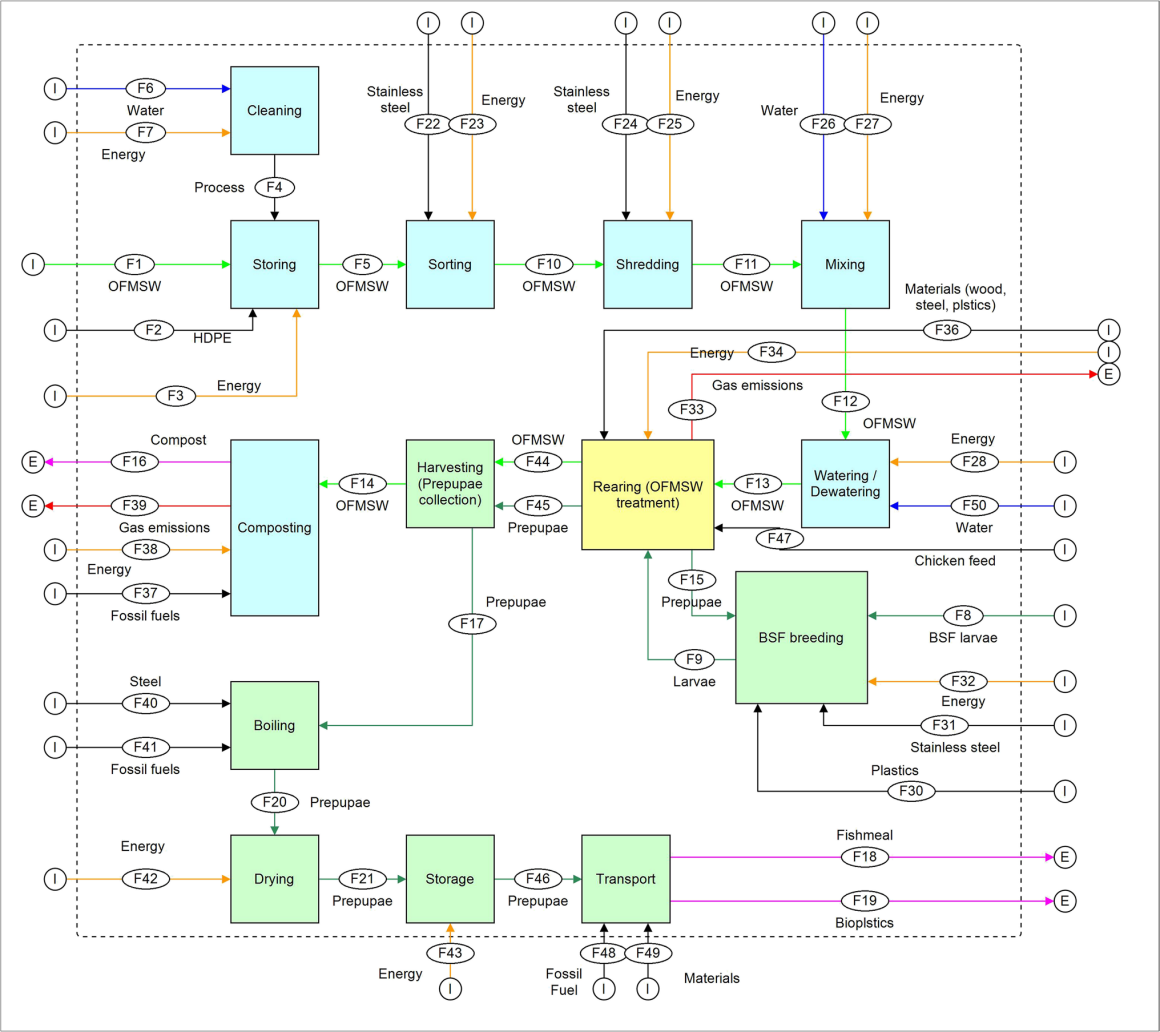
System boundaries. The blue boxes are related to OFMSW treatment processes, the green ones to BSF breeding and prepupae post-treatment, while the yellow ones refer to the core of the treatment process, where OFMSW is degraded and prepupae produced. The dashed line are system boundaries; E, outputs; I, inputs; Energy, electric energy; *F*(number): flow

The system boundaries of the study are reported in Fig. 2. Three steps can be identified: (i) waste pre-processing, (ii) waste treatment (BSF larval rearing), and (iii) products post-processing. The system begins with the raw OFMSW inflow into the plant. First, the feedstock is pre-processed and homogenized in terms of size and humidity. The research hypothesized that 100% of the material inflow into the system comes from a selective collection, and the inflow is 100% putrescible waste. Therefore, discarded materials from pre-treatments are not considered within the system boundaries. The OFMSW is then supplied as feedstock to the BSFL completing the larval cycle.

Two valuable products are obtained: the BSF prepupae, which undergo further post-treatment, consisting in boiling and drying, and the rearing residue, used for composting. Environmental benefits obtained in terms of impacts avoided are considered, such as the production of fishmeal, and the use of recyclable or biobased plastics for the production of mulching sheets, while the employment of fertilizers has not been included within the system boundaries. This has been omitted since compost is produced both in anaerobic digestion and composting and does not represent an added value to the process. Protein extraction from BSF prepupae and bioplastic production is outside the boundaries of the current research due to the lack of data. Results were compared with other conventional treatment options such as composting, anaerobic digestion, and final disposal.

### LCA - Life cycle inventory

The life cycle inventory was carried out by a screening and in-depth analysis of the information collected within the literature. In this way, the inventory for material depletion, energy consumption, water use, and feedstock conversion were assessed and implemented in the LCA model by the use of Ecoinvent databases. Avoided emissions were considered after evaluating the baseline environmental impacts through a scenario analysis. The electricity is assumed to be the average European consumption. About 25% of renewable energy is considered within the research in the baseline scenario, made of hydropower, wind power, and biomass, while the 75% comes from non-renewable energy. The complete electricity mix employed for the analysis is the following: 27% nuclear energy, 18.2% hydropower, 15.9% natural gas, 14.8% hard coal, 11.9% lignite, 4.6% wind, 2.4% biomass, 1.6% oil, and 3.6% others. The inventory is reported in the following chapters for each specific process. Finally, the land occupation indicator is assessed considering the information available within the Ecoinvent 3.5 database.

#### Waste pre-treatment

At this stage, the OFMSW is treated to make the feedstock optimal for BSFL. Details about waste characteristics are not reported since average values obtained by the literature were employed. Table 2 reports the inventory used for the LCA regarding this treatment phase. Material and energy consumption are introduced based on data collected from the literature. Storing phase consists of the location of the feedstock to be sorted. Here, the sorted and shredded biomass is collected to be treated by the BSFL. Sorting is mainly performed by a manual system with the support of a conveyor belt, while a shredding machine leads to size reduction. Materials employed for the system's operation are not considered within the impact analysis. Finally, mixing and dewatering were obtained with a mechanical system, while no wastewater is considered to be generated since it is assumed to be recirculated within the system to maintain the moisture content of the biomass. All mechanical systems are assumed to be electrical.

**Table 2:** Inventory analysis of OFMSW pre-treatment. Data are reported per FU (1 tonne of OFMSW)

Process	Description	Material consumption	Energy consumption	Source
Cleaning	Cleaning the units used during the processes	Water: 381 L t^−1^	Electricity (washing machine): 3.46 MJ t^−1^ Electricity:5.34 MJ t^−1^ (high-pressure cleaner)	Mertenat et al. (2019)
Storing	Waste holding containers with a capacity of 100 kg each, *h* 660 × *ø* 500 mm, thickness 5 mm, and density for polyethylene equal to 925 kg m^−3^; about 0.0015 m^3^ of polyethylene per unit are employed. Electricity consumption is for lighting and ventilation.	Total HDPE: 0.00762 kg (lifespan: 5 years)	Electricity (air conditioning): 9 MJ t^−1^	Material consumption (Dortmans et al. 2017), and energy consumption (Smetana et al. 2019).
Sorting	Conveyor belt. It is assumed to be made entirely of stainless steel. The selection is manual. Electricity consumption is required for moving the belt.	Stainless steel: 0.0548 kg (lifespan: 20 years)	Electricity: 0.005 MJ t^−1^	Data was collected from conventional types of machinery provided by private companies.
Shredding	The shredding system with a capacity of 1 t per hour. It is assumed to be made entirely of stainless steel.	Stainless steel: 0.0685 kg (lifespan: 20 years)	Electricity: 13.4 MJ t^−1^	Material consumption (Dortmans et al. 2017) and energy consumption (data from private companies)
Mixing, watering, and weighting	Mechanical mixing and weighting are assumed to be implemented. Watering is carried out in the function of the feedstock quality.	Water: 5 L t^−1^	Electricity: 0.072 MJ t^−1^	Water consumption (Smetana et al. 2019), and electricity consumption (Mertenat et al. 2019).

#### BSF breeding and waste treatment

The waste treatment consists in feeding the BSFL with the OFMSW. Table 3 reports the inventory employed for the analysis considering material use and energy consumption. On average, about 40.000 BSFL are required to treat 60 kg of organic substrate, needing at least 1 m^2^ of area (Smetana et al. 2019). Therefore, to treat about 1 t of OFMSW, 666,667 BSFL and about 17 m^2^ are required. This phase can be divided into two main processes:

- Insect breeding;
- BSFL rearing and OFMSW treatment.

**Table 3:** Inventory analysis of waste treatment: BSF breeding. Data are reported per FU (1 t of OFMSW). Data were obtained from Dortmans et al. (2017) and (Mertenat et al. (2019)

Process	Description	Material consumption	Energy consumption
*Love cage management*			
Love cage	Polyester mosquito-nets (3 units), 40 × 70 × 70 cm. The density of polyethylene is equal to 40 kg m^−3^. This value was used for all other polyester materials. It consists of 0.003 m^3^ of polyester.	Polyester: 1.8E−04 kg (lifespan: 5 years)	-
Water bowl	Polyethylene box (3 units), 30 × 40 cm. It consists of 0.0004 m^3^ of polyester per unit.	Polyethylene: 6.08E−04 kg (lifespan: 5 years)	-
Mobile Love-cage	Used to move the love-cage more easily, consisting of one stainless steel structure, 269 × 85 × 73 cm (single unit). A simple light bulb is used as an attraction light.	Stainless steel: 0.0018 kg (lifespan: 15 years)	Electricity (lighting): 0.216 MJ t^−1^
*Eggs and 5-days old larvae management*			
Hatching shower rack	The structure where the containers for pupate are located. Stainless steel structure, 165 × 141 × 62 cm (1 unit).	Stainless steel: 0.0027 kg (lifespan: 15 years) Drinking water: 0.17 L t^−1^	-
Hatching container	Polyethylene, 60 × 40 × 12 cm, for a total of 0.002 m^3^ of polyethylene per unit (8 units).	Polyethylene: 0.0081 kg (lifespan: 5 years)	-
Sieving system	With a mesh size of 1 mm, assumed made entirely of steel, 40 × 60 cm, for a total of 0.00024 m^3^ of stainless steel (2 units).	Stainless steel: 0.002 kg (lifespan: 5 years)	-
*Nursery management*			
Nursery container	Polyethylene, 55 × 35 × 16 cm, for a total of 0.0025 m^3^ of polyethylene per unit (7 units)	Polyethylene: 0.0089 kg (lifespan: 5 years)	-
Transfer container	Polyethylene containers (7units), 60 × 40 × 12 cm, totalling 0.002 m^3^ of polyethylene per unit.	Polyethylene: 0.0071 kg (lifespan: 5 years)	-
Nursery rack	Stainless steel, 155 × 141 × 62 cm. A weight of 12 kg per one is assumed (2 units).	Stainless steel: 0.0043 kg (lifespan: 15 years)	-
*Prepupae management*			
Dark cage	Polyester mosquito-net outside and dark soft fabric inside, 140 × 140 × 70 cm (3 units). It consists of 0.015 m^3^ of polyester and 0.029 m^3^ of dark soft fabric (density 450 kg m^-3^).	Polyester: 9.86E−04 kg (lifespan: 5 years) Dark soft fabric: 0.0215 kg (lifespan: 5 years)	-
Dark cage frame	Use to hang the dark cage (3 units), stainless steel structure 140 × 140 × 70 cm. A weight of 12 kg per one is assumed.	Stainless steel: 0.0065 kg (lifespan: 15 years)	-
Pupation container	The container where pupation takes place (43 units). Made by polyethylene, 60 × 40 × 12 cm, for a total of 0.002 m^3^ of polyethylene per unit.	Polyethylene: 0.043 kg. (lifespan: 5 years)	-

In particular, the breeding procedure can be divided in four sub-processes:

- Love cage management;
- Egg and 5-day-old larvae management;
- Nursery management.
- Prepupae management

The breeding procedure starts with mating BSF flies and laying eggs (i.e., love cage management). The system consists of a “love-cage,” with the addition of a substrate, generally characterized by a porous texture for egg laying while artificial light is adopted to simulate the solar one. The eggs, collected from the love cage, are placed in containers where hatching takes occurs. In the first 5 days of life, the newborn larvae must be treated with greater attention to allow a smooth development. At this stage, larvae are usually fed on chicken feed (5-day-old larvae management). For 1 kg of chicken feed, 0.12–0.36 MJ of energy is required in electricity and fuel, while the emissions are between 374 and 473 gCO_2_-eq (Usubharatana and Phungrassami 2016). After this period, the waste biomass is added into a single box, with a maximum quantity of 15 kg per unit (nursery management). The crates are then placed in piles of six containers each, to develop in height, limiting the space necessary for the plant. About 11 stacks are needed per tonne of OFMSW. The whole treatment usually requires 12 days. After this period, prepupae are removed and the rearing residue is gathered. Both outcomes of the process are sent to the post-treatment phase.

The process is considered to be completed after 12 days of treatment. BSF prepupae start to emerge spontaneously from the rearing residue; therefore, insects can be collected manually through a sieve (prepupae management). This method is not widely used on an industrial scale. This process leads to obtaining two products: BSF prepupae and the rearing residue. Table 4 reports the inventory analysis related to this process.

**Table 4:** Inventory analysis of waste treatment: rearing (OFMSW treatment) and harvesting (collection of prepupae). Data are reported per FU (1 t of OFMSW). Data were obtained from Dortmans et al. (2017) and Mertenat et al. (2019)

Process	Description	Material consumption	Energy consumption
*Rearing*			
Conversion crate	Polyethylene, capacity 15 kg each (67 units), 40 × 60 × 17 cm, thickness 5 mm. It consists of 0.0018 m^3^ of polyethylene per unit.	Inflow: Polyethylene: 0.061 kg (lifespan: 5 years) Chicken feed: 1.2 kg t^−1^ Outflow: CH_4_: 0.4 g t^−1^ CO_2_: 16 kg t^−1^ N_2_O: 8.6 g t^−1^	Electricity (climate system): 1.62 MJ t^−1^ Electricity (lighting): 7.92 MJ t^−1^
Pallets	Recycled wood, 800 × 1200 mm, for a single weight of 25 kg (11 units).	Wood: 0.151 kg (lifespan: 5 years)	-
Pallet trolley	A hypothetical weight of 79 kg for each pallet made up entirely of stainless steel (2 units) was assumed.	Stainless steel: 0.022 kg (lifespan: 20 years)	-
*Harvesting (prepupae collection)*			
Screens	Stainless steel screens, 40 × 60 cm (20 units). The density of stainless steel of 7600 kg m^−3^. This value was used for all other stainless-steel materials. It consists of 0.0005 m^3^ of stainless steel per unit	Stainless steel: 0.001 kg (lifespan: 10 years)	-
Harvesting containers	Polyethylene, capacity 100 kg each, 220 × *ø* 470 mm. It consists of 0.0009 m^3^ of polyethylene per unit (4 units). A high-pressure washer is employed for washing the entire area.	Polyethylene: 0.002 kg (lifespan: 5 years)	Electricity (pressurized water): 10.55 MJ t^−1^

#### Post-treatment

Post-treatment is implemented when the substrate is stabilized and the larvae become prepupae. About 1% of the prepupae obtained by the system is kept preserving the reproductive cycle. As the weight of a prepupa is about 200 mg, the total amount of prepupae obtained by the process can be estimated to be about 132 kg, while for 1 tonne of OFMSW, about 320 kg of treated waste can be gained (Vázquez and Soto 2017). The remain 99% of prepupae are sanitized, stored, and transported elsewhere for valorization. Sanitization can be performed by boiling prepupae in water for about 2 min to kill any pathogens. Then, depending on the product’s final destination, they can be stored or dried (Zurbrügg et al. 2018; Joly and Nikiema 2019). Table 5 reports the inventory related to the post-treatment phase.

**Table 5:** Inventory analysis of post-treatment processes. Data are reported per FU (1 tonne of OFMSW)

Process	Description	Material consumption	Energy consumption	Source
Disinfection stage (boiling)	Gas stove and cooking pot in stainless steel.	Stainless steel: 0.5 kg	Liquefied petroleum gas: 145.5 MJ t^-1^	Salomone et al. (2017); Mertenat et al. (2019); Smetana et al. (2019)
Drying	Electric oven employed for material frying.		Electricity (electric dryer): 5.76 MJ t^−1^	
Storage	Electric fridge for material harvesting.		Electricity (electric fridge): 39.74 MJ t^−1^	
Transportation	Transportation trucks for moving the outcomes of the process (BSF prepupae): Lorry 3.5–7.5 metric ton, EURO 6.	Maintenance: tiers, steel, and lubricants	Fossil fuel (diesel)	Ecoinvent 3.5 database
Composting	The composting process is hypothesized to be implemented near the BSF treatment unit.	Outflows: CH_4_: 630 g N_2_O: 63.3 g	Electricity: 2.02 MJ t^−1^ Fossil fuel (diesel): 9.68 MJ t^−1^	Mertenat et al. (2019)

Specific procedures for the extracting of proteins and fats can be performed, to obtain products with high economic value (bioplastics and biodiesel). The most abundant subproducts are lipids used for biodiesel production or other biotechnological applications (Saviane et al. 2021). Proteins are of high interest for the feed sector and bioplastics, while chitin has potential applications in the chemical and agronomical sectors (Leni et al. 2017). The residual part of the treated waste needs to mature before being used as compost. This can be done through a thermophilic and aerobic composting phase, which reduces the residual volume and the possible presence of further phytotoxicity and pathogens (Walker et al. 2009). While the composting process is only partly introduced within the LCA, the valorization of bioproducts is omitted due to the lack of data available in the literature. However, avoided emissions obtained, thanks to bioplastics or fishmeal production, are considered.

#### Avoided emissions

BSF prepupae can contain 32% protein and 9% chitin. Therefore, they represent a good starting point for producing bioplastics that can be potentially used for mulching (Alipour et al. 2019; Setti et al. 2020). Chitin is a polysaccharide representing one of the most abundant biopolymers in nature. Furthermore, thanks to its physic-chemical properties, it can be applied in different sectors such as biopharmaceuticals, textiles, food preservation, biocatalysts, wastewater treatment (Waśko et al. 2016), and 3D printed material (Sanandiya et al. 2020). Despite these good qualities, no evidence suggests using chitins from BSF prepupae to avoid any impact. Therefore, this research mainly focuses on the production of bioplastics from BSF proteins and the avoidance of plastic and fishmeal production.

BSF prepupae were assumed to substitute a fishmeal by a ratio of 1:2 (about 50%) since conventional fishmeal contains on average twice the protein content of larval meal on a wet weight basis. Therefore, a coefficient of 0.5 has been used to evaluate the avoided emissions. In particular, about 81.97 MJ of light fuel oil, 91.52 MJ of diesel, and 8.18 MJ of electric energy are required to produce conventional fishmeal and, therefore, can be avoided thanks to the use of BSF prepupae (Mertenat et al. 2019).

Regarding the production of bioplastics, the main application reports so far is the development of plastic films for mulching (Setti et al. 2020). They are usually produced from recycled polyethylene or bioplastics derived from starches. These materials already present in the Ecoinvent 3.5 inventory were chosen to perform the comparison. The bioplastic replacement rate from BSF prepupae is considered to be unlikely 1:1 due to the low quality of bioplastic from BSF. Therefore, a more conservative replacement rate of 0.8:1 has been used, representing the typical rate for recyclable plastics (Rigamonti et al. 2010).

### Results interpretation

For the interpretation of results, three different approaches were used:

- Sensitivity analysis, which able to identify the sensitivity of the final result to parameters fluctuation (Guo and Murphy 2012);
- Scenario analysis, which provides the different results that can be obtained due to the change in potential management scenarios (Steubing et al. 2016);
- Interval analysis, which gives optimistic and pessimistic environmental impact indicators in terms of the range of values obtained from the LCA modelling (Ziyadi and Al-Qadi 2019; Ferronato et al. 2020a).

The starting case, defined as scenario zero (S0), consists of a BSF treatment system with the same capacity as the FU (1 tonne of OFMSW per day). This choice might affect energy consumption and material balance. At this stage, avoided impacts are not considered. Then, five parameters were analysed, recognized as the most representative of the whole process (sensitivity analysis). Three possible situations were evaluated for each parameter to assess environmental impacts variability (scenario analysis). Finally, an interval analysis was carried out to estimate the best and worst scenarios that can be obtained from the scenario analysis conducted.

#### Sensitivity ratio

The sensitive ratio (SR) can be used to understand the sensitivity of the LCA model to a parameter change. Equation 1 reports the SR calculation, as suggested by (Clavreul et al. 2012):

1 $$\textrm{SR}=\frac{\frac{\Delta \textrm{result}}{\textrm{initial}\_\textrm{result}}}{\frac{\Delta \textrm{parameter}}{\textrm{initial}\_\textrm{parameter}}}$$

The equation evaluates the ratio between results and parameters relative changes. For example, if a parameter has a SR equal to 1, it means that when increasing its value by 10%, the final result increase by 10%, and can be concluded that the results are sensitive to the variation of this specific parameter. Therefore, this calculation allows understanding which parameter significantly influences the LCA’s final outcomes. The SR has been calculated for each chosen parameter.

#### Parameter’s assessment and scenario analysis

The studied parameters and the scenarios evaluated are reported as follows:

- Equipment lifespan. This parameter can considerably affect the final results of the analysis: 5, 10, and 15 years have been added to the starting standard data to see how the impacts could change, including a wider time frame of material consumption.
- Sub-product generation. It was checked how the results vary with fluctuations in production, which affect the impacts related to product transportation. Production of prepupae has been assessed first ranging from −10 to +10% and then in the presence of a much larger value equal to +50% (plant scale-up).
- Use of renewable energies. Electricity consumption can strongly affect the environmental footprint of the process. For this reason, it is assumed to employ renewable energy to quantify the environmental benefits. The energy demand input was changed using 50%, 75%, and 100% of electricity from renewable sources (solar energy).
- Energy consumption. The increase in treatment capacity might affect the energy consumption per functional unit. Higher capacity means lower energy consumption per FU. Therefore, 15%, 30%, and 50% decreased electricity consumption was considered.
- Transport of the final products. Goods transportation can affect the final results, particularly when traveling long distances. Analyses were carried out over distances of 25 km, 50 km, and 100 km, where 0 km is the starting parameter for S0.

Two additional scenarios were evaluated in terms of avoided impacts in parallel with the parameters’ assessment.

- Fishmeal substitution. As mentioned before, BSF prepupae can be used as fishmeal. The entire production chain of conventional fishmeal production is therefore avoided. In addition, three different transport scenarios were performed to consider the variability due to avoided transportation distances: On-site production (i.e., avoided transport equal to 0 km) has been compared with the production of fishmeal at the continental level, so with distances equal to 1000 km (lorry 3.5–7.5 metric ton, euro 6), and intercontinental production of fishmeal on routes of 10,000 km by cargo ship and a last hundred kilometres on the road.
- Bioplastics production. Recycled polyethylene or bioplastics deriving from starches were considered to be replaced by bioplastics produced from BSF proteins. Avoided road transportation equal to about 100 km was included.

#### Interval analysis and comparison with other case studies

The scenario analysis gives a higher and lower impact value for each environmental indicator and each management condition. For each parameter assessed, it can be possible to identify the best condition for obtaining a positive effect on the environment or the worst one for increasing the environmental footprint of the process. Therefore, for quantifying the best general management condition involving all operational parameters, the best positive variations (negative values) related to each scenario (parameter analysis) were collected for each environmental indicator. Then, they were added to the impacts obtained for S0. The calculation gives a final output showing the minimum potential impact generated by the system. Similarly, the same analysis can be conducted for the worst management condition.

The results were compared with the literature. First, the final range of value was compared with the previous LCA of OFMSW treatment with BSFL to validate the results of the LCA model implemented in this research. Then, having a range of values (interval analysis), the results were compared with other treatment options, such as OFMSW disposal into sanitary landfills, composting, and anaerobic digestion from Mondello et al. (2017). Refer to the scientific article for the system boundaries and general characteristics of the LCA carried out for the conventional treatment option (Mondello et al. 2017). Thus, it is possible to evaluate which of the treatment described can be potentially the one with the most benefits from an environmental point of view and understand which factors can lead to critical issues.

## Results

### Result normalization

From the IMPACT 2002+ method normalization, the impact categories mainly affected by the system were identified. All values are expressed as eco-points (namely mPt). The scale represents 1 Pt for one-thousandth of the yearly environmental load of one average European inhabitant. This inhabitant value is obtained by dividing the total environmental load in Europe by the number of inhabitants and multiplying it by 1000 (scale factor). The analysis identified six mid-point categories as the most relevant detectable within the system boundaries.

**Fig. 3:**
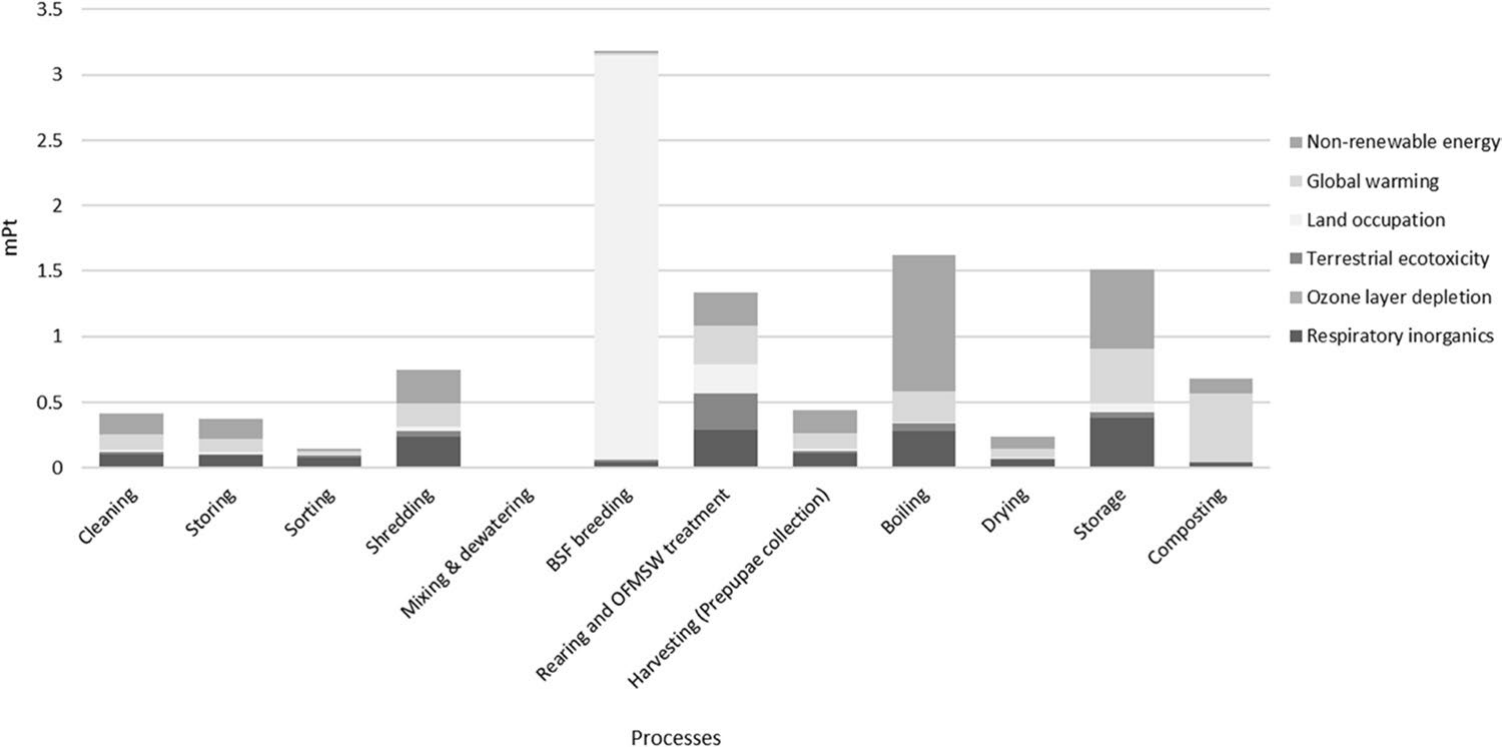
Normalization of the results per environmental impact indicators (mid-point categories)

The impact category that presents a relatively high environmental burden is land occupation, directly linked to the BSFL breeding process, global warming, respiratory inorganics, and non-renewable energy. On the other hand, aquatic acidification and eutrophication are equal to zero since this impact category is directly related to pollutants released in water bodies not generated by the system (Jolliet et al. 2003). Six main environmental impact categories can be identified as the most contributing parameters to environmental impact: respiratory inorganics (kg PM2.5-eq), ozone layer depletion (kg CFC-11-eq), terrestrial ecotoxicity (kg TEG soil), land occupation (m^2^ organic arable), global warming (kg CO_2_-eq), and non-renewable energy (MJ primary). Figure 3 shows the normalized environmental impacts for each process. It can be underlined that the most relevant unit process generating impacts is BSF breeding (land occupation), followed by boiling, storage, and OFMSW treatment.

### Contribution analysis

**Fig. 4:**
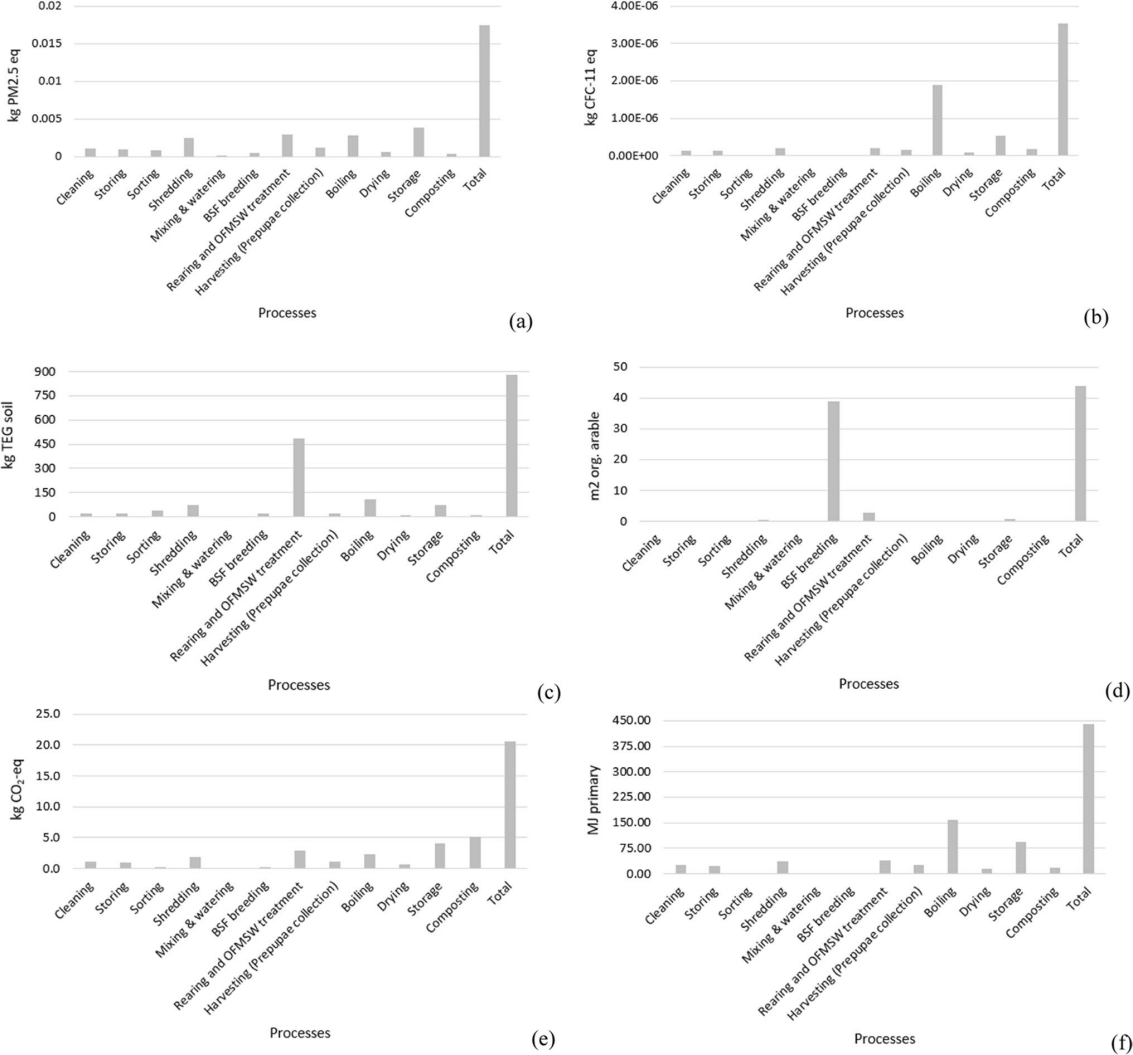
Contribution analysis of the LCA per environmental impact indicator: **a** respiratory inorganics potential; **b** ozone layer depletion potential; **c** terrestrial ecotoxicity potential; **d** land occupation potential; **e** global warming potential; **f** non-renewable energy

The results at mid-point categories after the characterization per treatment process are reported in Fig. 4. Here, the environmental impact indicators are described based on the specific unit measurement (or indicator) which characterizes a certain mid-point category. Contribution analysis is shown for the most relevant environmental impact categories identified by results normalization. By the analysis, the environmental impact indicators can be quantified and the contribution of each process to the indicators can be defined.

Concerning respiratory inorganics, a total of 17.45 E−03 kg PM2.5-eq are generated per FU. The main contribution derives from storage, boiling, rearing and breeding, and shredding systems. These processes relate to the impacts due to the consumption of electric energy produced mainly by fossil fuels. Similarly, the ozone layer depletion is due to the consumption of fossil fuels, largely employed during the sterilization procedure (boiling) and storage, due to energy consumption for air conditioning and amounts to 3.50E−06 kg CFC-11-eq. Terrestrial ecotoxicity is mainly due to BSF larval rearing and OFMSW treatment. This indicator is mainly affected by the use of chicken feed, for a total impact of about 850 kg TEG soil. The breading unit mainly contributes to land occupation, affecting more than 90% of the total impact, equal to about 44 m^2^ organic arable land. This is due to the space required for larval breeding (material consumption and storage), while the carbon footprint is mainly linked to the composting stage, followed by the processes that mainly consume electric energy and fossil fuels, such as storage, boiling, rearing, and shredding.

### Sensitivity ratio

**Fig. 5:**
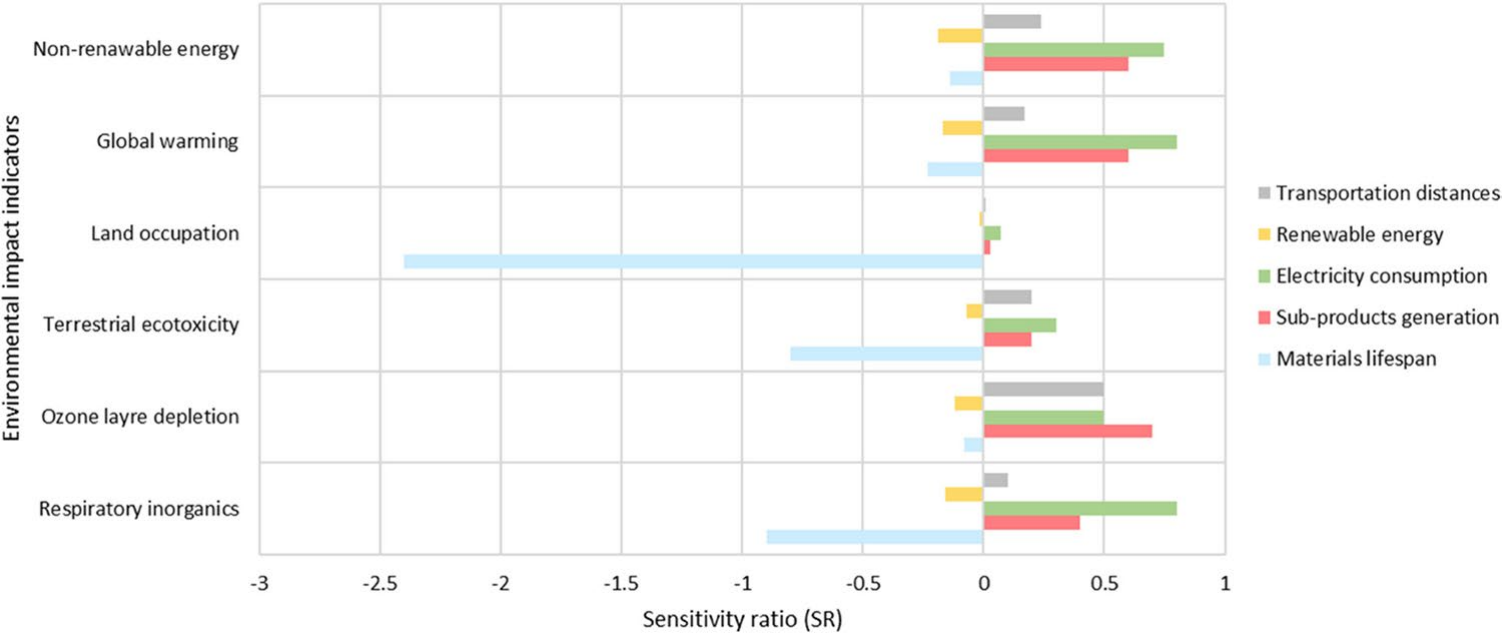
Sensitivity rate (SR) for the most relevant environmental impact indicators

The SR has been calculated for each parameter that can potentially affect the results of the LCA. Figure 5 reports the outcomes of the analysis. The most sensitive parameter (SR > 1) is related to the material lifespan of land occupation. It means that 10% changes in the material lifespan are reflected in a land occupation decrease of about 24%, contributing to modifying the final result of the LCA considerably. This is due to the strong correlation between material consumption and land occupation within the whole material life cycle.

Other moderately sensitive parameters refer to the electricity consumption (i.e., 1 < SR < 0.5). This is mainly related to non-renewable energy, global warming potential, ozone layer depletion, and respiratory inorganics. Similarly, the increase of sub-products generation, which directly influences the transportation process, also affects the same EII, except respiratory inorganics. Therefore, increasing energy consumption and sub-products generation by about 10%, these EII increase of a similar percentage, from 5 to 10%. Furthermore, transportation distances affect the ozone layer depletion results (SR equal to 0.5). Finally, renewable energy use does not considerably affect the result since SR is always lower than 0.5. On balance, the parameters that mostly affect the results are materials lifespan, electricity consumption, and sub-products generation.

### Scenario analysis: including avoided emissions

Prepupae obtained by the system can potentially be employed as fishmeal. The avoided impacts obtained, thanks to the avoidance of conventional fishmeal production and transportation, has been calculated assuming three different scenarios: (i) replacing the fishmeal with no transport avoided, (ii) avoiding the road transportation for 1000 km, and (iii) a mixed sea road transport for 10,000 km. Results are reported in Table 6. The substitution of fishmeal production allows for reducing all EII. In particular, ozone layer depletion is reduced by 78%, while non-renewable energy of about 54%. Of the three scenarios, the best results are avoiding fishmeal production 1000 km from the production site. In particular, ozone layer depletion is reduced by 246%, terrestrial ecotoxicity by 227%, and global warming by about 175%. Finally, the last scenario, where fishmeal is produced 10,000 km far from the site, allows achieving higher impact avoidance and the lower EII related to respiratory inorganics, equal to about 183%. This outcome suggests that impact avoidance within the LCA considerably allows for mitigating the impacts generated by the OFMSW treatment by BSFL.

**Table 6:** Comparison of EII considering the avoidance of fishmeal production and bioplastics and recycled plastics production (FU: 1 t of OFMSW). The percentages in brackets represent results variation compared to S0

Scenarios	Respiratory inorganics	Ozone layer depletion	Terrestrial ecotoxicity	Land occupation	Global warming	Non-renewable energy
	kg PM2.5-eq	kg CFC-11-eq	kg TEG soil	m^2^ org. arable	kg CO_2_-eq	MJ primary
S0	0.0174	3.50E−06	879	44	20.62	439
Fishmeal avoided 0 km	0.0137	7.63E−07	738	43.7	17.9	202
	(−21.3%)	(−78.2%)	(−16.0%)	(−0.7%)	(−13.2%)	(−54.0%)
Fishmeal avoided 1000 km	-0.006	−5.11E−06	−1117	38.8	−15.5	−331
	(−134.4%)	(−246%)	(−227.1%)	(−11.8%)	(−175.2%)	(−175.4%)
Fishmeal avoided 10,000 km	−0.0144	−8.12E−07	454	43	8.43	64.6
	(−182.8%)	(−123.2%)	(−48.4%)	(−2.3%)	(−59.1%)	(−85.3%)
Avoidance of bioplastics production	0.0171	3.45E−06	875	41.3	20.4	432
	(−1.7%)	(−1.4%)	(−0.5%)	(−6.1%)	(−1.1%)	(−1.6%)
Avoidance of recycled polyethylene production	0.0174	3.49E−06	872	43.9	20.5	438
	-	(−0.3%)	(−0.8%)	(−0.2%)	(−0.6%)	(−0.2%)

The proteins extracted from the prepupae can also be valorized in bioplastics. The hypothesis is that bioplastics from BSF can substitute bioplastics or recycled plastics used for mulching sheets. The outcomes of the analysis show that the avoidance in bioplastics or recyclable plastics are not environmentally relevant. Benefits are in fact obtained, but they are not comparable with fishmeal production. Within the simulation of the variability related to the avoided transportation of bioplastics production, it was found that variation equal to about 6% can be reached by doubling the avoided transportation distances. This demonstrates that the avoidance of transportation distances does not considerably affect the results related to the avoidance of bioplastics or recycled plastics production.

### Interval analysis

**Fig. 6:**
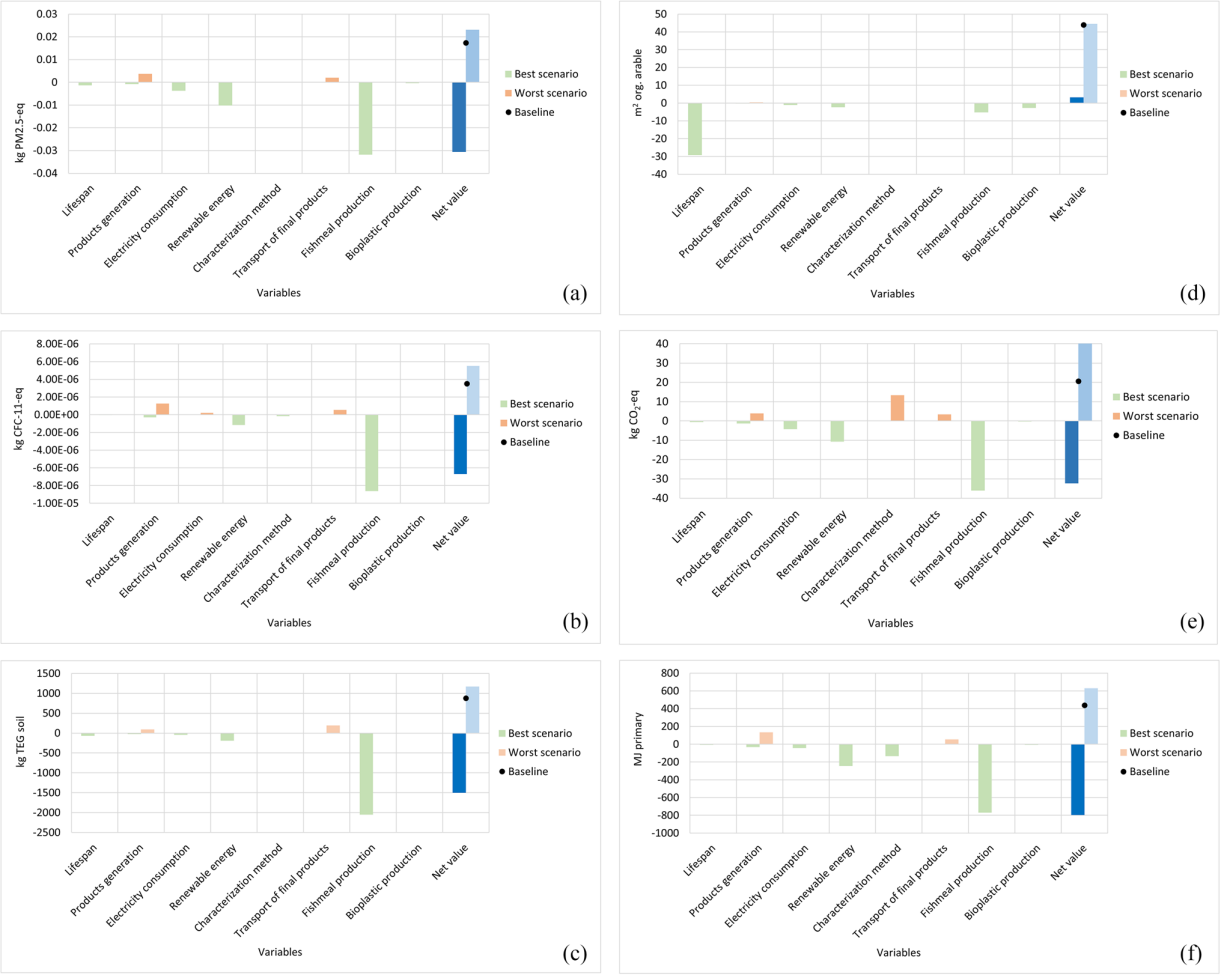
Interval analysis per environmental impact indicator: **a** respiratory inorganics potential; **b** ozone layer depletion potential; **c** terrestrial ecotoxicity potential; **d** land occupation potential; **e** global warming potential; **f** non-renewable energy. The light blue bar represents the maximum impact (worst scenario), and the blue bar represents the lowest (best scenario)

An interval analysis evaluated the maximum and minimum values of the worst and best scenarios. Figure 6 reports the min and max values related to each EII and each assessed variables. The green bars represent the net environmental gains that can be obtained by the introduction of the most favourable scenario, while the orange bars define the net environmental losses that are achieved in the case that the worst condition has been considered within the model. The total gives the net minimum and maximum impact that can be obtained by the LCA, indicating the variability that can be identified through the study.

Summarizing, global warming ranges from -32.4 to +41.1 kg CO_2_-eq, respiratory inorganic from −0.031 to +0.0231 kg PM2.5-eq, ozone layer depletion from −6.7E−6 to +5.5E−6 kg CFC-11-eq, terrestrial ecotoxicity from −1499 to 1169 kg TEG soil, land occupation from 3.2 to 44.5 m^2^ org. arable, and non-renewable energy from −797 to 629 MJ primary.

High variability can be observed. The system can obtain positive environmental effects if avoided impacts are considered. The baseline scenario has a pessimistic condition since the avoided impacts are not taken into account and renewable energies are not implemented. On balance, the best scenario can be defined if the fishmeal produced with conventional systems derives from areas 10,000 km far, the system employs 100% renewable energy, the energy consumption is reduced by 50%, the lifespan of the equipment increase of 15 years, the products are employed nearby the system, and the most favourable impact assessment method is used (IMPACT 2002+). These are the main characteristics that can be detected to obtain higher benefits from the OFMSW treatment system. This result can be compared with other case studies to validate the results’ magnitude.

## Discussion

### Limitations of the LCA and considerations about the results

The LCA herein presented allows comparing the environmental impacts generated by OFMSW treatment with BSFL. The limitation of the analysis is due to the use of secondary data for the inventory analysis. In addition, the inventory analysis related to composting is quite limited, making the final results sensitive to a variation in this process that can considerably influence the results (Mancini et al. 2019). Although avoided impacts due to the employment of compost as alternative fertilizer were not considered, balancing the limited estimations related to the composting process. Similarly, procedures for bioplastics production from larval proteins were not assessed. This is due to the lack of data within the literature. It is another essential practice that should be evaluated and assessed within future LCA to make the results more reliable. It also involves treatment processes that are not estimated in the current research but are relevant for obtaining better results (Bishop et al. 2021). It is recommended that future researches focus on data collection associated to biobased materials produced from BSF.

On the other hand, the environmental benefits of OFMSW treatment with BSFL can be affected by some parameters that can reduce the advantages obtained from the valorization of BSF products. First, transportation distances of the final products can increase the environmental impacts of the system (Ferronato et al. 2022). Longways for transporting BSF bioproducts can increase the environmental impacts, affecting the positive environmental results related to the impacts avoided thanks to the process. Therefore, OFMSW treatment plants should be located close to industries capable of employing BSFL as secondary raw material. Second, energy consumption is relevant, and it affects the results. In particular, air conditioning is one of the most critical processes influencing the system. The use of renewable energy (i.e., solar panels) should be considered to mitigate the effects of global warming, increasing the systems’ benefits. Finally, material lifespan considerably influences all environmental impact indicators. Material consumption is high during the process: for example, the system requires the employment of chicken feed (or similar) and plastic boxes and nets, among others, that can have a short lifecycle. Therefore, alternative long-term materials should be considered within the system.

Data employed for the inventory of the LCA came from different literature and databases located in different countries. It means that energy and material consumption, production, and depletion efficiency data are affected by different climatic conditions, organic waste characteristics, and process flows, among other parameters. For example, composting emissions can vary significantly. Therefore, it should be underlined that all these variabilities can affect the results and a “real-world situation” should be carefully evaluated to better compare OFMSW treatment options. However, the results provide a range of values that can be assumed to be the higher and lower impacts that the process can obtain at a global level.

### Comparison with other studies and treatment options

Comparisons with other LCA conducted by previous studies are reported in Table 7. It can be highlighted that the current research employed the IMPACT 2002+ characterization method and considers various environmental impact indicators together with Salomone et al. (2017). The range of global warming and ozone layer depletion values is coherent with other studies, except for energy consumption. Nevertheless, the presentation of the results in terms of the range of values provided a considerable advance in LCA assessment, providing the spectrum of the best and worst scenario that can be obtained in the function of different parameters.

**Table 7:** Comparison with the literature related to LCA results

Environmental impact indicators	Unit	Mertenat et al. (2019)	Mondello et al. (2017)	Salomone et al. (2017)	Current research	
					Min	Max
Global warming	kg CO_2_-eq	35	35.02	24.584	−32.39	41.42
Respiratory inorganic	kg PM2.5eq				−0.0306	0.0231
Ozone layer depletion	kg CFC-11eq		4.02E−6	1.4E−6	−6.7E−6	5.5E−6
Terrestrial ecotoxicity	kg TEG soil				−1499	1169
Land occupation	m^2^ org. arable				3.2	44.55
Non-renewable energy	MJ primary		772		−797	629

The same results can be compared with conventional treatment options. Data for the comparison were obtained from the literature (Mondello et al. 2017), which used CML 2 baseline 2000 method. Results are reported in Table 8. It can be underlined that global warming seems to be always lower than sanitary landfill and composting, also in the worst condition. However, anaerobic digestion looks to provide better results if compared with analysis’s maximum value (worst condition). Similar results were obtained for ozone layer depletion, while non-renewable energy consumption is better for BSF treatment than conventional treatment options, achieving a maximum value that is 10 times lower than composting processes. On balance, BSF seems to have similar environmental impacts compared to anaerobic digestion, achieving considerable advantages if the most optimistic condition is considered.

**Table 8:** Comparison of the BSF with conventional treatment options per FU (Mondello et al. 2017)

	Unit	Sanitary landfill	Composting	Anaerobic digestion	BSF	
					Min	Max
Global warming	kg CO_2-_eq	1146–1243	59–99	−299–66	−32.39	41.42
Ozone layer depletion	kg CFC-11eq	3.04E−05	7.75E−06	5.03E−06	−6.7E−06	5.5E−06
Non-renewable energy	MJ	5236	875	−1658–982	−797	629.24

### Research developments and policy implications

Efforts should be spent to find alternative applications of BSF to increase the added value of the final product. For example, the quality of the bioplastics produced can be investigated to make bioplastics for higher-level uses. At the same time, protein extraction processes and chemical reactions able to collect lipids and chitins should be carefully considered within an LCA. For example, regarding protein extraction, it has been estimated that an enzymatic approach resulted 31.9% more impactful compared to the chemical method, and advanced biomass pre-treatment or different proteolytic enzymes are required (Rosa et al. 2020). Furthermore, future research in biotechnology for the valorization of chitins, lipids, and proteins can be explored to increase the opportunities to replace fossil fuels or plastic-based goods (Smets et al. 2020).

The LCA of full-scale treatment plants with primary data can be beneficial (Ferronato et al. 2023). It is recommended to start implementing pilot plants to isolate data and evaluate the feasibility of exploiting BSF for industrial purposes. For example, the avoided impacts related to fishmeal productions cannot always be employed since BSF prepupae obtained from OFMSW treatment cannot be always used as alternative fishmeal although it depends on the regulatory bases. In fact, since 2017, the European Commission Regulation 2017/893 has moderately lifted the ban rules regarding the use of processed animal proteins from insects, so it is now possible to include BSF in feeds for aquaculture. However, in both cases, feedstuff containing BSF proteins can be formulated exclusively using insects that have been reared on selected and safe waste substrates.

Research should also focus on characterizing nutrient variability of waste and developing efficient and timely collection and transport systems (Rajeh et al. 2021). This issue can affect the positive results related to fishmeal production from the OFMSW treatment from a legal point of view. Prepupae should be identified as a by-product from the regulations to move to an end-of-waste policy. On the other hand, results obtained in our analysis suggested that OFMSW treatment by BSFL can be considered a viable and promising alternative treatment option instead of conventional treatment plants. Moreover, if implemented with renewable energy, BSF can be a tangible alternative. Therefore, national regulations should move towards investing in these treatment alternatives to minimize the waste inflow into final disposal sites, increase waste valorization opportunities, and produce valuable biobased materials focusing on a circular bioeconomy (Leong et al. 2021).

## Conclusions

The current research comprehensively studies the environmental impacts generated by a BSF treatment process. The results show the most important EII and the most affected processes. Six out of fifteen environmental impact categories are considered the most relevant (respiratory inorganics, ozone layer depletion, terrestrial ecotoxicity, land occupation, global warming, and non-renewable energy).

BSF breeding, boiling, storage, and OFMSW treatment are the most relevant processes generating impacts, all requiring high energy consumption, which is the most important factor affecting the results. All indicators perform better if the substituted fishmeal produced with conventional systems derives from areas 10,000 km far, the system employs 100% renewable energy, the energy consumption is reduced by 50%, the equipment’s lifespan increases by 15 years, and the products are employed nearby the treatment plant. The most sensitive parameter is material lifespan concerning land occupation, while electricity consumption is the most relevant parameter associated to non-renewable energy, global warming potential, ozone layer depletion, and respiratory inorganics.

The final output of the research provided indications about the range of values that can be obtained for the most important EII: global warming (−32.4 to +41.1 kg CO_2_-eq), respiratory inorganic (−0.031 to +0.023 kg PM2.5-eq), ozone layer depletion (−6.7E-6 to +5.5E−6 kg CFC-11-eq), terrestrial ecotoxicity (−1499 to +1169 kg TEG soil), land occupation (3.2 to 44.5 m^2^ org. arable), and non-renewable energy (−797 to +629 MJ primary). Comparing these results with conventional treatment options demonstrates that BSF treatment performers are potentially better than final disposal and composting, while it seems to perform better than AD, although not in the worst process conditions.

In conclusion, the study demonstrates that OFMSW treatment using BSFL can be an environmentally attractive option compared to traditional treatment plants. If renewable energy is employed and low electricity consumption is achieved, BSFL can be a good alternative to support sustainable OFMSW treatment. Primary data about proteins extraction and bioplastics production are required, together with full-scale treatment plants, giving the LCA more relevant and reliable information. Future research should focus on this side to better support the sustainable application of this technology towards a circular bioeconomy.

## Data Availability

The datasets generated during and/or analysed during the current study are available from the corresponding author on reasonable request.

## Nomenclature

LCA: Life Cycle Assessment
SWM: Solid waste management
MSW: Municipal solid waste
BSF: Black Soldier Fly
BSFL: Black Soldier Fly Larvae
EII: Environmental impact indicators
FU: Functional unit
OF: Organic fraction
OFMSW: Organic fraction municipal solid waste
SR: Sensitivity ratio
GWP: Global warming potential
